# A “Hit and Run” Approach to Inducible Direct Reprogramming of Astrocytes to Neural Stem Cells

**DOI:** 10.3389/fphys.2016.00127

**Published:** 2016-04-12

**Authors:** Maria Poulou, Nikolaos P. Mandalos, Theodoros Karnavas, Marannia Saridaki, Ronald D. G. McKay, Eumorphia Remboutsika

**Affiliations:** ^1^Stem Cell Biology Laboratory, Biomedical Sciences Research Centre “Alexander Fleming,”Vari-Attica, Greece; ^2^Choremio Laboratory, Department of Pediatrics, National University of Athens Medical SchoolAthens, Greece; ^3^Basic Sciences Division, The Lieber Institute for Brain DevelopmentBaltimore, MD, USA

**Keywords:** FLP, tetracycline, doxycycline, CRE^ERT2^, embryonic stem cells, induced pluripotent stem cells, neural progenitor cells, tissue regeneration

## Abstract

Temporal and spatial control of gene expression can be achieved using an inducible system as a fundamental tool for regulated transcription in basic, applied and eventually in clinical research. We describe a novel “hit and run” inducible direct reprogramming approach. In a single step, 2 days post-transfection, transiently transfected Sox2^FLAG^ under the Leu3p-αIPM inducible control (iSox2) triggers the activation of endogenous Sox2, redirecting primary astrocytes into abundant distinct nestin-positive radial glia cells. This technique introduces a unique novel tool for safe, rapid and efficient reprogramming amendable to regenerative medicine.

## Inducible gene expression systems

From the time of the first transcriptional regulatory systems (Gossen and Bujard, 1992), several inducible gene expression systems were developed (Clackson, 1997; Saez et al., 1997; Rossi and Blau, 1998) for applications in gene function analysis (Malleret et al., 2001), drug discovery (Aubel et al., 2001), gene therapy (Auricchio et al., 2002), engineering of desired phenotypes during development and in adult life (Niwa et al., 2000), trangenesis (Rossant and Nagy, 1995), stem cell programming and reprogramming (Brambrink et al., 2008; Hockemeyer et al., 2008; Maherali et al., 2008; Stadtfeld et al., 2008, 2010; Welstead et al., 2008; Wernig et al., 2008; Markoulaki et al., 2009; Carey et al., 2010). Regulatory systems developed to control the temporal and spatial gene expression use mostly fusion proteins as regulators and hormones or antibiotics as signals for gene expression. In mammals, the most commonly used inducible systems include the tetracycline system (Sprengel and Hasan, 2007), the systems of the recombination enzymes Cre/loxP (Sauer, 1998) and Flipase/FRT (Hummel and Klämbt, 2008), and the CRE-ER^T2^ system based on the function and modularity of the ligand-binding domain of the estrogen receptor (ER)(Chiba et al., 2000). The “OFF/ON” gene switches allow for the expression of dominant negative and cytotoxic proteins (Angrand et al., 1998), for the reversibility of the target gene expression (Kistner et al., 1996), for the study of “loss or gain” of function phenotypes (Caulin et al., 2007) and for the isolation of functional components (Meissner et al., 2003). The limitations of these systems lie within the fact that antibiotics are used as regulators of gene expression, resulting in cytotoxicity, interference with embryonic development, the high cost of the inducer, leakiness, chromosomal alterations, immune response and incompatibility in the integration into the regulatory and metabolic network of the target cell (Danielian et al., 1998; Gao et al., 1999; Loonstra et al., 2001; Wunderlich et al., 2001). Thus, tighter control of gene induction with no side effects is fundamental for gene function analysis, for the development of animal models and most importantly for the development of novel approaches in gene and stem cell therapy.

## Leu3p-α-IPM inducible gene expression

We have previously reported a novel heterologous ligand-inducible regulatory “OFF-ON” genetic switch, based on the yeast transcription factor Leu3p (Leu3p-α-IPM; Poulou et al., 2010). This system is based on a transcription factor (Leu3p) involved in the regulation of the leucine pathway in yeast, whose function is controlled by α-IPM, a metabolite involved in leucine biosynthesis itself (Kohlhaw, 2003). Leu3p acts as an active repressor binding to its UAS_LEU_DNA element, turning into an activator of the transcription in the presence α-IPM (Sze et al., 1992), an ideal inducer since it exhibits lipid solubility, metabolic stability, rapid “OFF-ON” kinetics with no apparent toxicity to mammalian cells, to fertilized mouse eggs cultured *ex vivo* and to animals alike (Poulou et al., 2010). Although the leucine biosynthetic pathway is found only in prokaryotes, fungi and plants, Leu3p has been shown to be fully functional In mammalian cells in culture (Sze and Kohlhaw, 1993; Remboutsika, 1994; Guo and Kohlhaw, 1996) and in primary mouse embryonic fibroblasts isolated from double transgenic mouse embryos bearing ubiquitously expressing Leu3p and a Leu3p regulated GFP reporter (Poulou et al., 2010). This system would be ideal for use in stem cell programming and reprogramming strategies transferable from the bench to the clinic.

## Present study

Here, we show for the first time that the Leu3p-α-IPM inducible system can be used in direct reprogramming experiments.

## Aim

To directly reprogram astrocytes to neural stem cells by “hit and run” Leu3p-α-IPM inducible *Sox2* expression approach.

## Methods

### Experimental animals

*Sox2*^*COIN*∕+^ mice were bred with Tg(*hGFAP*:CRE) mice (Zhuo et al., 2001) to generate *Sox2*^*RG*−*INV*∕+^ mice harboring an ablation of Sox2 function in radial glia cells. All animals were handled in strict accordance with good animal practice as defined by the Animals Act 160/03.05.1991 applicable in Greece, revised according to the 86/609/EEC/24.11.1986 EU directive regarding the proper care and use of laboratory animals and in accordance to the Hellenic License for Animal Experimentation at the BSRC” Alexander Fleming” (Prot. No. 767/28.02.07) issued after protocol approval by the Animal Research Committee of the BSRC “Alexander Fleming” (Prot. No. 2762/03.08.05).

### Primary astrocyte culture

Brains were carefully dissected from P3 mouse pups, the meninges were removed and the remaining brain tissue was digested with 0025% trypsin in CaMg-free HBSS. The tissue was pipetted up and down with a 10 ml pipette to generate a single cell suspension, shaken for 10 min at 37°C on a rotary shaker, in a waterbath (5 ml per brain). At the end of the incubation time, 5 ml of DMEM containing 10% FBS were added, the sample was pipetted a few times with a 10 ml pipette and passed through a 70 μm nylon mesh to remove the debris. After centrifugation at 200 g for 10 min, the cell pellet was washed once in complete media before the cells were re-suspended in 7 ml complete media, plated out in T 25 cm plates coated poly-L lysine (100 mg/ml). The astrocytes were allowed to grow to confluency (~10–12 days) with media changes every 3 to 5 days. P3 astrocytes were cultured at a density of 2 × 10^4^ cells/cm^2^ in 4 well Sonic Shield culture dishes (Nunc-Thermo Fisher Scientific, Roskilde, Denmark). Transfection was performed using Lipofectamine 2000 (Invitrogen) according to the manufacturer's instructions. α-IPM (20 mM final concentration) was added to the culture media 1 day post-transfection. Cells were incubated for another 24 h, rinsed with 1 × PBS, fixed in 4% ice-cold PFA in 0.12 M PB for 10 min on ice, rinsed again with 1 × PBS multiple times, before use for immunofluorescence or mounting in 50% glycerol before phase contrast photography on a Leica DMI3000 microscope.

### RT-PCR

Total RNA was isolated using TriZol, according to the supplier's instructions, (Invitrogen) and RT-PCR was carried out using the Qiagen One-Step RT-PCR system. The following primers were used: for *Gapdh* (T = 57°C): 5′-CATCTCTGCCCC CTCTGCTG-3′ (forward) and 5′-CGACGCCTG CTTCACCACCT-3′ (reverse); for endo*Sox2* (T = 62°C): 5′- CCCCCGGCGGCA ATAGCA -3′ (forward) and 5′-TCGGCGCCG GGGAGATACAT-3′ (reverse); *for Sox2*^*FLAG*^ (T = 60°C): 5′-CCCCCGGCGGCAATA GCA-3 (forward) and 5′-TCAAAG CTTGTCATCGTCGTCCTT-3′ (reverse); for *wnt3a* (T = 52°C) 5′- ATTGAATTTGGA GGAATGGT-3′ (forward) and 5′- CTTGAAGTA CGTGTAACGTG-3′ (reverse); for *nestin* (T = 60°C) 5′- CGCTGGAACAGAGAT TGGAAGG-3′ (forward) and 5′ –GTCTCAAGG GTATTAGGCAAG- 3′ (reverse).

### Immunohistochemistry

Cells and cortical sections were fixed in 4% PFA in 0.12 M PB, pH 7.2 at 4°C for 5 min and incubated in Blocking buffer (BB; 0.12 M PB, pH 7.2, 0.15% glycine, 2 mg/ml BSA fragment V (Gibco-Invitrogen) and 0.1% Triton X-100) for 1 h on ice. Cells were incubated o/n at 4°C with primary antibodies in BB. After extensive washes with PBS at RT, cells were incubated with species specific secondary antibodies (Alexa 488-conjugated, 1:500; Invitrogen) for 1 h at RT. Samples were mounted in anti-fade DAPI mounting media (Invitrogen) and photographed on a Leica SP5 confocal microscope. Primary antibodies: anti-Nestin (mouse IgG 1:75, Developmental Studies Hybridoma Bank (DSHB), University of Iowa, http://dshb.biology.uiowa.edu), anti-RC2 (mouse IgM 1:100, DSHB), anti-GFAP, Cy3-linked (mouse IgG 1:100, SIGMA), anti-Sox2 (rabbit IgG 1:1000, Santa Cruz, INC).

## Results and discussion

**Figure 1:**
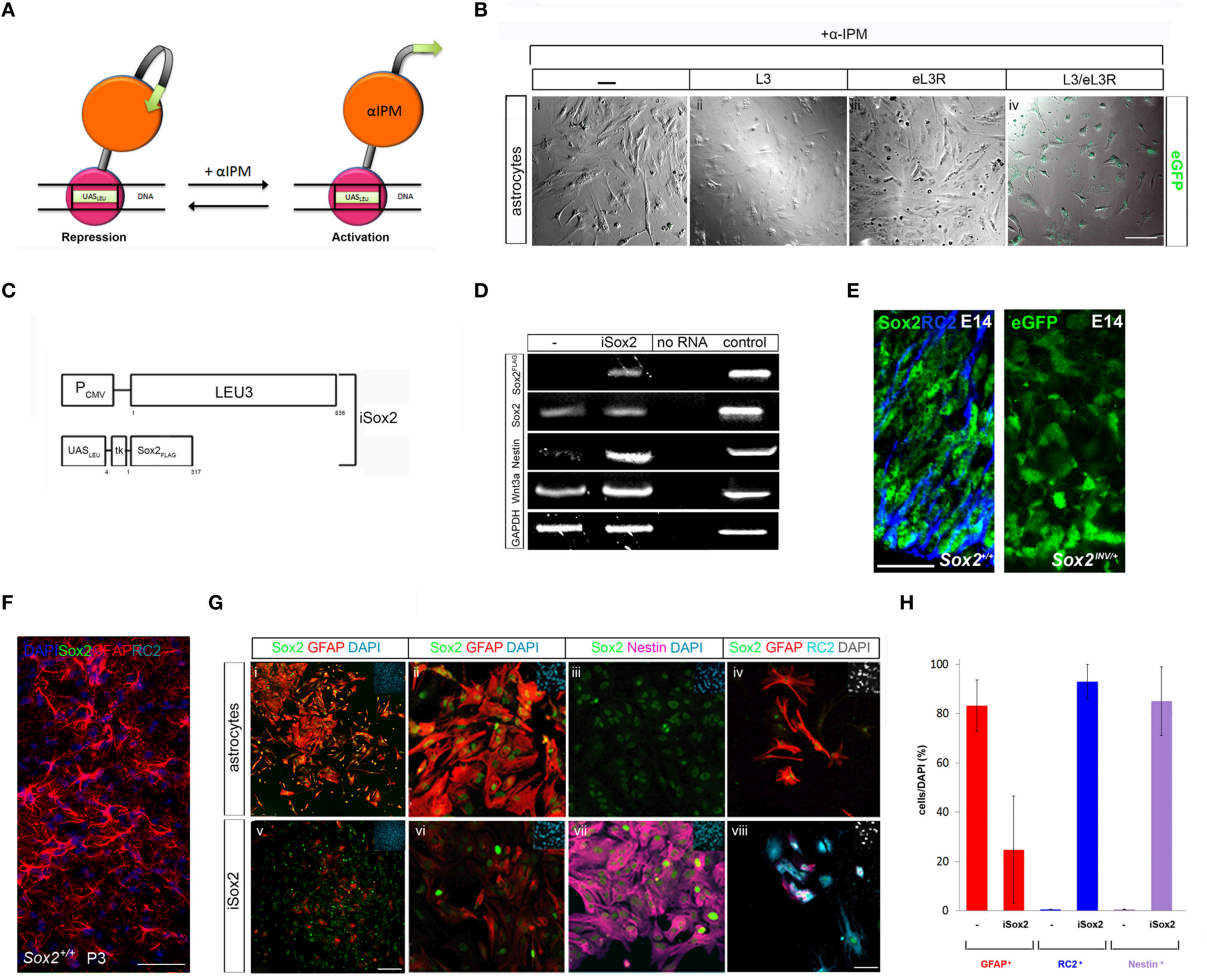
**Leu3p-α-IPM inducible fast - track direct reprogramming of astrocytes to neural stem cells. (A)** Leu3p·α-IPM mode of action: Transcriptional repressor upon binding to the UAS_LEU_ DNA element and transcriptional activator upon α-IPM ligand binding. **(B)** Superimposed bright field and confocal images of transiently transfected primary P3 murine astrocytes (i-iv) with either Leu3p protein (L3) (ii) or UAS_LEU_-eGFP reporter (eL3R) (iii) or both L3/eL3R (iv). eGFP is observed only in cells UAS_LEU_ with both L3/eL3R (iv). Scale bar 75 μm. **(C)** Generation iSox2 expression system under the control of Leu3p UAS_LEU_ elements. **(D)** *iSox2* induces endogenous *Sox2, nestin*, and *wnt3a* 48 h post-transfection in P3 primary murine astrocytes. **(E)** Sox2^+^ and RC2^+^ neural progenitors in the proliferating zone of E14 cortex of wild type mouse embryos. eGFP expression detected in the proliferating zone of E14 cortex after *Sox2* ablation in ragial glia cells. Scale bar 75 μm**. (F)** Sox2 is not expressed in GFAP^+^ cells in the proliferating zone of P3 mouse cortex. Scale bar 50 μm. **(G)** iSox2(v-viii) reduces the astrocytic marker GFAP (i,-ii, iv–vi) in P3 primary murine astrocytes and induces a Nestin^+^ (vii) radial glia (RC2^+^) (viii) NSC phenotype 48 h post-transfection. Dapi staining of nuclei is present in the upper right corner in all panels. Scale bars for panels i and v represent 250 μm and for panels ii-iv and vi-viii represent 75 μm. **(H)** Graphs depicting the GFAP^+^ and either nestin^+^ or RC2^+^ cells in untransfected astrocytes and in reprogrammed cultures.

Nervous stem repair is an enormous challenge to regenerative medicine. Even though, neural stem/progenitor cells (NSCs) can be generated as an intermediate cell type from astrocytes on their way to generate neurons *in vivo* and *in vitro*, limitations including viral infection and integration, as well as uncontrolled reactivation of transgenes hamper their use in clinical applications (Tomanin and Scarpa, 2004; Huang and Tan, 2015). Here, we investigated the efficiency of the Leu3p-α-IPM as a “hit and run” gene switch to generate NSCs from astrocytes *in vitro*. We first demonstrated that α-IPM addition efficiently induced a Leu3p regulated eGFP reporter in transiently transfected primary astrocytes (Figures 1A,Bi–iv). NSCs develop and maintain their properties under the guidance of Sox2 (Ferri et al., 2004; Remboutsika et al., 2011; Mandalos et al., 2014; Figure 1E) and they can be generated by direct lineage reprogramming of astrocytes via *in vivo* overexpression of Sox2 (Niu et al., 2013). We then designed an iSox2 Leu3p inducible system to test the rapidness and efficiency in imposing Sox2 reprogramming ability to primary astrocytes *ex vivo* (Figure 1C). Although, *in vivo* at P3 astrocytes do not appear to express Sox2 (Niu et al., 2013; Figure 1F), in primary astrocyte cultures *Sox2* (Figure 1D) and Sox2 protein (Figure 1Gi–iv) are expressed in low levels. Astrocytes do not express Nestin (0.6 ± 0.1%) or RC2 (0.5 ± 0.1%), markers of NSCs with a radial glia character (Figure 1D,Gi–iv). Two days post-transfection, high levels of Nestin (85 ± 13.89%) and RC2 (93 ± 6.97%) are expressed only in cells after Sox2 induction (iSox2) in astrocyte cultures (Figures 1D,Gv-viii,H). Concurrently, GFAP^+^ cells are reduced in iSox2 cultures (24.8 ± 21.73%; Figures 1Gv-viii,H), respectively, when these are compared to control astrocyte cultures (83.28 ± 10.43%; Figures 1Gi-viii,H). One would expect that only the cells with the highest expression of Sox2 could revert the astrocytic (GFAP^+^) to a NSC (Nestin^+^, RC2^+^) radial glia phenotype. However, we observed that the majority of cells have acquired a Nestin^+^ phenotype. This led us to believe that the iSox2 cells now express a signaling molecule that controls NSC identity. One of these could be *wnt3a*, known to be involved in the proliferation of NSCs (Lie et al., 2005), but also expressed in low levels in untransfected astrocytes cultures (Figure 1D). Indeed, *wnt3a* was induced in iSox2 NSCs, along with the induction of endogenous *Sox2* and *nestin* (Figure 1D). Thus, iSox2 can generate NSCs from astrocytes rapidly and efficiently *in vitro*.

To build an ideal regulatory system for clinical research three factors are important (a) activation by a highly specialized non-toxic bio-available exogenous ligand, (b) inactivation in the absence or removal of the ligand, and (c) no interference with endogenous mammalian gene expression and metabolic pathways. Evidently, Leu3p-α-IPM represents this novel unique tool for simple, safe, fast and efficient inducible programming and reprogramming in different cell types for gene and stem cell strategies directly amendable to the clinic.

## Ethics statement

All animals were handled in strict accordance with good animal practice as defined by the Animals Act 160/03.05.1991 applicable in Greece, revised according to the 86/609/EEC/24.11.1986 EU directive regarding the proper care and use of laboratory animals and in accordance to the Hellenic License for Animal Experimentation at the BSRC“Alexander Fleming” (Prot. No. 767/28.02.07) issued after protocol approval by the Animal Research Committee of the BSRC “Alexander Fleming” (Prot. No. 2762/03.08.05).

## Author contributions

MA, NM, TK, MS conducted research experiments; RM provided reagents; ER directed research and wrote the manuscript.

## Funding

This research was financed by the Studentship Grant PENED 394, the European Union (ERDF) and Greek National funds through the Operational Program “Competitiveness and Entrepreneurship” of the NSRF (Cooperation Grant-09∑YN9D-12-966), the ADISC (THALIS) MIS380249 Grant funded by Framework Program “Education and Lifelong Learning,” co-financed European Social Fund and National funds (Ministry of Education) and a collaboration grant (NKUA-12439) from the Lieber Institute for Brain Development to E.R. (US).

### Conflict of interest statement

The authors declare that the research was conducted in the absence of any commercial or financial relationships that could be construed as a potential conflict of interest.
